# Digital Therapeutic Content for Substance Use Disorder Treatment: Development and Evaluation Study

**DOI:** 10.2196/87453

**Published:** 2026-05-07

**Authors:** Nicholas L Bormann, Alyssa Kalata, Stephan Arndt, Tyler S Oesterle

**Affiliations:** 1 Department of Psychiatry and Psychology, Mayo Clinic, 200 1st St SW, Rochester, MN, 55905, United States, 1 507-284-2511; 2 Department of Psychiatry, University of Iowa, Iowa City, IA, United States; 3 Department of Biostatistics, University of Iowa, Iowa City, IA, United States

**Keywords:** mobile health, mHealth, therapeutic content, usability, digital therapeutics, patient-centered care, mobile apps, addiction

## Abstract

**Background:**

Substance use disorders (SUDs) are a major public health concern, contributing to significant individual and societal costs. Despite this, the uptake of evidence-based pharmacologic and behavioral interventions remains limited. The digital delivery of SUD treatment has emerged as a potentially scalable way to reduce access barriers and increase treatment use. Existing digital therapeutic interventions are often created without clinician involvement, evidence-based materials, interdisciplinary input, or content review. The implementation of a structured and methodologically rigorous development process is needed across digital health interventions to help ensure patient-facing materials are validated, understandable, and actionable for the end user.

**Objective:**

This early report seeks to describe and evaluate an iterative, interdisciplinary, platform-agnostic process for adapting and refining existing print materials for digital therapeutic modules in SUD treatment. The a priori goal was to evaluate if a structured, human-centered approach would generate digital modules that were rated as understandable and actionable based on a validated assessment for written materials.

**Methods:**

Fourteen therapeutic modules were adapted from existing Mayo Clinic–written, patient-facing education materials originally developed by a board-certified addiction psychiatrist and a doctoral-level education specialist for clinical use. A team of 4 purposively recruited licensed alcohol and drug counselors with lived experience with a SUD, all in recovery, and a doctoral-level therapeutic specialist met weekly for one hour over a 6-month period to iteratively adapt this existing content for smartphone delivery (2‐3 hours per module). The process flow included selecting source material, restructuring content for viewing on a phone screen, simplifying language, improving organization and flow to promote understanding, and including specific actions users could take based on the content. The counselors then independently evaluated the modules using the Patient Education Materials Assessment Tool for printable materials (PEMAT-P). PEMAT-P scores for understandability and actionability were calculated as percentages, and descriptive statistics were used to summarize scores in aggregate and across modules. A target of >70% was set for each PEMAT-P domain, consistent with accepted benchmarking standards.

**Results:**

Mean understandability and actionability for all modules were 87.2% (SD 4.8%; range 81.4%‐96.9%) and 75.1% (SD 12.3%; range 57.1%‐95.0%), respectively, exceeding the recommended threshold. While all modules were adequately understandable, 35.7% (5/14) scored below the actionability threshold.

**Conclusions:**

This early report highlights the value of a human-centered, iterative process for adapting therapeutic materials for digital delivery in SUD treatment. Although the modules performed well overall on PEMAT-P benchmarks, actionability was less consistent than understandability, and aggregate scores masked weaknesses in several individual modules. This indicates that a standardized process does not guarantee actionable material across all content types. Involving current patients in this process may improve the end product by incorporating a perspective that was previously missed.

## Introduction

Substance use disorders (SUDs) affect over 48 million people in the United States, yet treatment use remains strikingly low [1]. In 2022, only 24% of individuals needing treatment accessed inpatient, outpatient, or telehealth services [1]. The use of evidence-based medications was even lower, with just 20% of adults receiving these for opioid use disorder and 1.6% for alcohol use disorder [2,3]. Persistent barriers such as stigma, geographic inaccessibility, and workforce shortages contribute to these care gaps [4-6]. Mobile health (mHealth) apps have emerged as a promising way to help address these challenges by offering a low-barrier, scalable mechanism for delivering therapeutic content directly to patients’ smartphones [7].

mHealth app creation, however, is varied and often limited in transparency [8-10]. Prior reviews have identified important limitations in health and substance use–focused mHealth apps, including minimal involvement of health care professionals, little to no evidence-based therapeutic content, and a lack of validation for usability or clinical effectiveness [8-10]. Basic informational summaries and general recommendations predominate rather than actionable strategies that patients can use [8,10]. Furthermore, understandability (likelihood that a reader can grasp and explain key messaging) and actionability (likelihood that a reader will recognize and then act on specific information) are rarely assessed [11]. Poorly designed materials may also fail to account for varying levels of health literacy, leaving users confused or unsupported and reducing the likelihood that digital interventions are usable, engaging, or beneficial [10,12-14].

By incorporating end users into the development process, aspects of these content limitations may be improved. Human-centered design leverages the lived experience and knowledge of patients to better match the intervention to the motivations, needs, life constraints, and overall context that app users may find themselves in when accessing the intervention [15]. Iteration, including developing, testing, evaluating, and refining content based on user feedback, is a foundational aspect of human-centered design [15,16]. Modules are well suited for this iterative improvement, allowing for specific parts of a larger intervention to be optimized based on targeted feedback [16]. Engagement with digital content is often low [14], and evidence suggests that a lack of the end user’s perspective in development stages contributes to this issue [17]. Past reviews note that end users are rarely involved throughout all aspects of digital intervention creation, particularly in the overall design stage [17,18]. By including individuals with lived experience in this process, the acceptability and engagement with digital interventions have shown benefit [18,19].

Past work has incorporated elements of human-centered design in digital interventions for SUD treatment, but this is limited. In one study, focus groups were conducted iteratively with a group of women in recovery from various SUDs. Their feedback guided mHealth app feature development, leading to greater emphasis on housing, counseling, and parenting advice. However, there were no structured assessments of understandability or actionability [20]. A pilot study of military veterans with problematic alcohol use repeatedly assessed the attractiveness and usability of an mHealth app focused on alcohol use reduction. However, they were not involved in its creation or development, nor did they assess the therapeutic content itself [21]. Outside of substance use, study evaluators concluded that apps for chronic health conditions contained few actionable targets for behavior change and were below accepted thresholds for understandability and actionability [22]. Therefore, while aspects of human-centered design have been applied in related settings, the literature on SUD-focused digital interventions provides limited information on how therapeutic content is adapted for mobile delivery and evaluated through structured assessments of understandability and actionability [23].

To help address this gap, iterative approaches that involve key stakeholders in the development and evaluation of mHealth app–based therapeutic content are needed. This includes transparency in how materials are adapted for digital delivery, how understandability and actionability are assessed, and how the content is honed over time. In this early report, we describe how an interdisciplinary team adapted, refined, and evaluated therapeutic modules for delivery within a mobile digital platform for SUD treatment [24,25]. We expected that an iterative development process would produce modules that met established benchmarks for understandability and actionability.

## Methods

### Study Design

This project was an iterative content creation and evaluation study conducted as part of an internal program development effort focused on producing therapeutic materials used in an mHealth app–based intervention for SUD treatment [24-26]. The purpose was to describe and evaluate a careful, step-by-step process for how existing clinical content could be adapted for smartphone delivery and whether the end product met measurable standards for understandability and actionability.

### Participants and Recruitment

Licensed alcohol and drug counselors (LADCs) employed by Mayo Clinic within the Division of Addiction Medicine (Department of Psychiatry and Psychology) were recruited to participate. They worked with a doctoral-level therapeutic specialist, employed within the same department. The a priori recruitment goal was 4 LADCs, which was felt to be a reasonable number to balance group discussion with the doctoral-level therapeutic specialist.

Approximately 15 LADCs are employed within the division at any given time. Consistent with their field broadly, a significant number of these providers are themselves openly in recovery from a SUD [27]. This background provides lived experience and a point of shared connection with their patients, a perspective sought out for this study. Our division has a strong history of patient- and staff-oriented addiction research. As such, a group of approximately 8 LADCs exists who have actively participated in previous projects. Four of these counselors, known to be openly in recovery from a SUD, were purposively recruited by email. Participation was entirely voluntary. The LADCs could freely decline to participate, as they had done previously when they were not interested in a project. Declining to participate would have no impact on their job.

The first 4 LADCs approached all agreed to participate. These specific LADCs were asked due to their experience working in multiple levels of addiction treatment, including level of care assessments, individual therapy, group-based outpatient groups, and residential programming, and their previous involvement in research within the division. Collectively, the participating LADCs had decades of experience working with individuals with SUDs and co-occurring mental health diagnoses and were themselves openly in recovery from a SUD. Their typical work roles include providing individual and group-based SUD treatment, performing care coordination on their patient’s behalf, and helping complete patient-related paperwork. Module review occurred in blocked times during their standard work hours. The doctoral-level therapeutic specialist performed this role as part of their typical duties within the department.

### Content Development Process

Fourteen therapeutic modules were developed from existing Mayo Clinic text-based materials originally created by a board-certified addiction psychiatrist and a doctoral-level education specialist for use in clinical practice. These materials required reformatting for a smartphone interface and needed to be easy to read, visually clear, and to use everyday language.

#### Iterative Revision

The module adaptation process occurred over 6 months by an interdisciplinary team of LADCs with lived experience in recovery and a doctoral-level therapeutic specialist. The team met weekly for 1-hour meetings, spending approximately 2 to 3 hours on each module. The following process occurred for each module:

- Selection of source material: an existing therapeutic topic was identified from Mayo Clinic patient educational content used in current clinical practice.
- Content restructuring: the layout and flow of information required changing to allow visualization on a screen compared with a physical handout. This was accomplished by breaking longer passages into shorter sentences, creating more subheadings to help direct eye movement and orient to the screen size, and simplifying verbiage to approximately a fifth-grade reading level. The doctoral-level education specialist helped to balance clarity and brevity, trying to ensure messaging was understandable. Content sought to provide clear guidance while avoiding being overly directive or containing lecturing language.
- Actionability: the source content was often primarily informational. Therefore, specific and feasible steps an end user could realistically take based on the information provided were included in every module. They were drawn from the LADCs’ combined clinical expertise and personal experience, reflecting their dual roles as health care providers and individuals in recovery from a SUD [27].
- Team-based review: module drafts were discussed in a nonhierarchical way, with the previous steps being repeated to allow for revision. Once group consensus was obtained, the modules were prepared for digital conversion and eventual formal assessment.

#### Evaluation

Once finalized, the LADCs independently evaluated the modules on their own smartphones, using the Patient Education Materials Assessment Tool for printable materials (PEMAT-P) [28]. This validated instrument assesses module materials across 2 domains: understandability (17 items) and actionability (17 items). Understandability reflects the likelihood that readers can grasp and explain the material’s key messages. It evaluates clarity of language, organization, and visual presentation, including whether the material uses everyday language and whether sections have informative headers. Actionability reflects the likelihood that readers will recognize and subsequently be able to take specific actions based on the information presented. This includes assessing whether the material clearly identifies at least one user action and whether recommended steps are listed in manageable components [28].

Each item was scored as agree (1), disagree (0), or not applicable. Domain scores were calculated as percentages by dividing the number of agree responses by the total number of scored items (ie, zero or one). For each module, mean scores and SDs were calculated across raters, consistent with prior research and recommendations [28]. Modules scoring below the commonly recommended 70% threshold were identified as areas that may need further revision.

### Ethical Considerations

The home institution considered this project to be quality improvement in nature and therefore determined it exempt from review by the Mayo Clinic Institutional Review Board. Staff participated voluntarily and were given dedicated time during their regular workday to complete the study tasks. No patients were involved, and no patient information was used.

## Results

**Figure 1.**
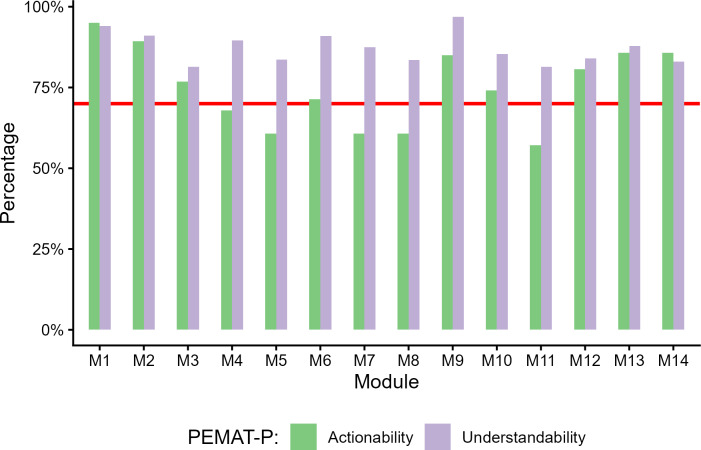
Module-level quality assessment of digital therapeutic content. Mean understandability and actionability scores for 14 therapeutic modules evaluated using the Patient Education Materials Assessment Tool for printable materials (PEMAT-P). Modules: M1 (Introduction), M2 (Managing Thoughts), M3 (Thinking Errors), M4 (Addictive Thinking), M5 (Recovery Thinking), M6 (Replacing Old Thoughts with New), M7 (Identifying Feelings and Emotions), M8 (Experiencing Emotions), M9 (Self-Esteem and Self-Talk), M10 (Relationships), M11 (Triggers), M12 (Coping with Cravings), M13 (Self-Nurturing Skills), and M14 (Safety Planning and Relapse Prevention). Bars represent mean scores for each module across 4 independent raters. The horizontal red line represents the recommended PEMAT-P threshold of 70% for understandability and actionability.

Table 1 displays the title, brief description, and mean understandability and actionability for each module. The 14 modules in aggregate demonstrated high overall performance on the PEMAT-P. Mean scores across all modules were 87.2% (SD 4.8%; range 81.4%‐96.9%) for understandability and 75.1% (SD 12.3%; range 57.1%‐95.0%) for actionability. The highest scoring modules included Introduction (understandability: 94%; actionability: 95%) and Self-Esteem and Self-Talk (understandability: 96.9%; actionability: 85%). Modules with lower actionability scores (<70%) included Recovery Thinking, Replacing Old Thoughts with New, Identifying Feelings and Emotions, and Triggers, with actionability ranging from 57.1% to 60.7%. Figure 1 illustrates the understandability and actionability scores across modules.

**Table 1.** Included modules^a^.

Module	Description	Understandability (%), mean (SD)	Actionability (%), mean (SD)
Introduction	Provides a foundational understanding of addiction to ensure all engaging have a specific level of knowledge in preparation for subsequent modules	94.0 (4.8)	95.0 (10.0)
Managing thoughts	Focuses on how thought patterns can influence one’s actions, including substance use, and begins to build skills to manage these more effectively	91.1 (17.9)	89.3 (21.4)
Thinking errors	Focuses on identification of inaccurate or unhelpful thinking patterns to support more balanced decision-making	81.4 (21.8)	76.8 (27.0)
Addictive thinking	Aims to increase recognition of maladaptive thought patterns associated with substance use to reduce the risk of returning to use	89.6 (14.2)	67.9 (47.2)
Recovery thinking	Supports the development of rational, realistic, recovery-oriented thinking, including mapping out long-term behavior change and how that supports the goal of recovery	83.7 (19.0)	60.7 (48.6)
Replacing old thoughts with new	Helps users identify previous distorted or unhelpful thoughts and replace them with more grounded and adaptive beliefs that can support them in their goals	90.9 (18.2)	71.4 (48.1)
Identifying feelings and emotions	Connects how recognizing and naming emotions and feelings can lead to a better understanding of their effect on substance use and eventual recovery	87.5 (25.0)	60.7 (48.6)
Experiencing emotions	Supports recognition and processing of emotions commonly experienced during recovery to strengthen emotional awareness and coping skills	83.5 (19.2)	60.7 (48.6)
Self-esteem and self-talk	Works to normalize how self-esteem can be low early in recovery and that self-talk can fluctuate between supportive and antagonistic, with an aim of improving awareness of how individuals view themselves	96.9 (6.3)	85.0 (30.0)
Relationships	Focuses on understanding the impact of substance use on past and current relationships and how to build healthier sources of mutual support	85.4 (17.6)	74.1 (35.4)
Triggers	Helps users identify internal and external triggers of cravings and how these can impact returns to substance use	81.4 (21.8)	57.1 (50.8)
Coping with cravings	Builds on identified triggers to establish skills for recognizing, tolerating, and managing cravings	84.0 (18.6)	80.7 (26.9)
Self-nurturing skills	Encourages the identification, development, and regular practice of individualized self-care practices that promote well-being	87.8 (16.7)	85.7 (28.6)
Safety planning and relapse prevention	Supports development of practical plans and coping strategies to maintain safety and help mitigate relapse; specific, concrete steps that can be taken if sudden issues occur are emphasized	83.0 (20.5)	85.7 (28.6)

a The titles, descriptions, module-level mean ratings (n=4 reviewers) of understandability and actionability for 14 therapeutic mobile app modules developed for a digital platform for substance use disorder treatment. The Patient Education Materials Assessment Tool was adapted to evaluate whether therapeutic modules met established quality thresholds (≥70%).

## Discussion

### Principal Results

This early report described the iterative development and evaluation of 14 therapeutic modules for an mHealth app–based SUD treatment platform and examined whether they met recommended PEMAT-P benchmarks (>70%) for understandability and actionability. On aggregate, the modules exceeded both understandability (87.2%) and actionability (75.1%) targets, supporting the use of an interdisciplinary and iterative approach. Despite this resource- and time-intensive process, several modules fell below the actionability benchmark, which may indicate that our methodology was more effective for understandability or that understandability itself is easier to achieve than actionability. Additionally, these findings highlight the challenges of achieving consistent quality even when a structured process is implemented and suggest that process improvement is needed to maximize understandability and actionability for the end user.

### Comparison With Prior Work

Descriptions of how existing therapeutic content is adapted for digital delivery remain limited, particularly in SUD interventions, which also lack systematic evaluations of understandability and actionability [23]. This gap exists for chronic health conditions more broadly, where digital interventions (n=44) have been found to include few actionable targets for behavior change and to fall below accepted thresholds for understandability and actionability (medians of 42% and 0%, respectively) [22].

Our modules were found to have better understandability than actionability. This distinction has also been seen in past literature. In bipolar disorder, a review of online resources (n=52) found that most scored poorly on the PEMAT-P, with mean understandability of 56.0% (SD 14.0%) and mean actionability of 44.0% (SD 24.0%) [29]. Similarly, an evaluation of psychiatric hospital brochures found a mean PEMAT-P score of 48.4% (SD 15.4%) and noted that nearly all materials were written at a grade 12 or higher reading level [30]. Reported deficiencies included insufficient emphasis on key information, unexplained medical terminology, limited guidance on recommended behaviors, and visuals that were either irrelevant or too complex to enhance understanding [30]. Finally, problematic PEMAT-P scores have also been observed outside of mental health treatment. A review of online materials for rotator cuff repair reported mean understandability and actionability scores of 64.6% and 29.5%, respectively, with no identified material surpassing the actionability threshold [31].

Across these studies, a common thread is the influence of health literacy on whether patients can actually use the material in a meaningful way. Defined as an individual’s ability to obtain, process, and understand basic health information needed to make appropriate health decisions, health literacy is a critical determinant of equitable health care delivery [32]. Deficits in health literacy are pervasive, contributing to poorer outcomes and higher health care use [32]. Therapeutic materials that fail to meet health literacy standards can widen existing disparities by limiting access to information, reducing patient engagement, and undermining informed decision-making. Our process involved a doctoral-level therapeutic specialist to maintain the material at a fifth-grade reading level. Ensuring that educational and therapeutic content are understandable and actionable is essential for supporting patient engagement and helping individuals achieve their treatment goals [28,32].

In this context, using the above-described strategy, our tested modules compare favorably with mean understandability and actionability scores exceeding those reported in prior studies. The SDs observed for modules were variable, and at times, quite large. Looking at Table 1 and Figure 1, it is apparent that module-level differences exist. Differences may have also occurred at the reviewer-level; however, this study was not designed to compare the relative contribution of scale item, rater, and module effects. Information on this is also missing from the existing literature [23]. Prior work with the PEMAT-P suggests that expanding the number of independent reviewers in future evaluations may provide more robust estimates of content quality [33].

### Considerations for Future Development and Evaluation

A structured, interdisciplinary approach can raise the quality of digital therapeutic materials, but content quality alone does not fully explain how users experience or benefit from them. Importantly, our study assessed content quality rather than perceived patient benefit. High PEMAT-P scores indicate that users should be able to grasp and act on the material. Conversely, modules scoring lower may still be perceived as useful by some users for reasons that lie outside the PEMAT-P’s scope. This interpretive gap is not unique to our project but reflects a broader challenge in digital health evaluation [34]. Known as the digital placebo effect, this phenomenon refers to improvements in outcomes or perceived benefit that arise from assuming an intervention will be useful because the content is medical in nature [34]. For example, in a study of healthy participants using an inert mHealth app, being told prospectively or retrospectively that the intervention would improve mood and stress was associated with higher credibility and expectancy ratings compared with a control condition [35].

While the therapeutic value of mHealth apps depends primarily on the quality of their content and how they are delivered [7,14], the digital placebo effect suggests that users’ expectations can be shaped by the way an intervention is framed [34,36]. As such, future research should prioritize rigorous development and evaluation of both content and delivery strategies.

**Figure 2.**
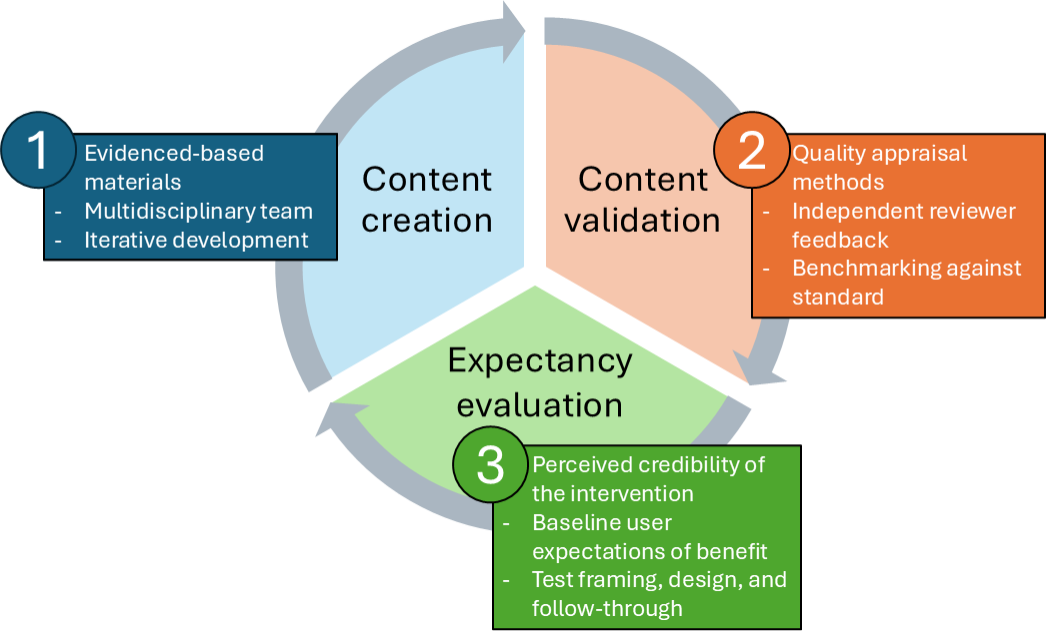
Iterative framework for creating, validating, and evaluating digital therapeutic content. Developing digital therapeutic content requires an iterative and multilayered approach. Step 1: Content Creation involves adapting evidence-based materials through an interdisciplinary team and refining content with iterative development. Step 2: Content Validation applies structured appraisal tools, such as the Patient Education Materials Assessment Tool, to evaluate understandability and actionability, with feedback guiding revisions. Step 3: Expectancy Evaluation addresses the digital placebo effect by examining user credibility ratings, baseline expectancy measures, and testing the influence of design and framing features. Insights from both content validation and expectancy evaluation inform subsequent design cycles.

Several steps are essential to advancing mHealth app–based research that directly assesses the efficacy of therapeutic content (Figure 2). First, incorporating parallel expectancy scoring alongside PEMAT-P ratings may help identify gaps between content quality and perceived credibility. For example, counselors could provide a brief expectancy rating (eg, “How likely is this module to feel credible or motivating to patients?”). Second, introducing brief patient expectancy probes in patient-facing pilots, such as a single premodule item (eg, “How helpful do you expect this module will be?”), could quantify baseline expectations and help separate content effects from potential placebo effects in follow-up data. Third, incorporating automated follow-up action checks may provide an observable behavioral end point to compare with PEMAT-P’s actionability scores. For instance, modules that conclude with behavioral activation activities could be paired with an mHealth app–delivered push notification 1 hour later asking whether the assigned task was accomplished. Finally, iterative A/B feature testing could evaluate how variations in framing, design cues, or reminders impact user follow-through. Collectively, these steps may help provide a more accurate representation of the mHealth app’s effect.

### Limitations

This project has several limitations. The number of LADCs involved was small (n=4) and purposively recruited, which may have introduced selection bias and limited the range of perspectives represented. Additionally, this group of LADCs helped both create and review the material. This level of investment may have added bias, such as contributing to score inflation. A group of independent LADC evaluators may have added a layer of objectivity. While these counselors were in recovery from SUDs and had extensive knowledge and training in SUD treatment, their ratings may not reflect how patients with varying literacy levels, lived experiences, referral sources, or differing trajectories of addiction severity and treatment responsiveness may interpret the material [37,38]. Including participants with SUDs who were not health care professionals would have strengthened the human-centered design aspect of module evaluation. Additionally, the PEMAT-P captures important aspects of quality, but it does not measure perceived credibility, motivation, or expectancy, which may influence how users engage with therapeutic content. The modules were adapted from existing clinical materials rather than created de novo, which may have influenced how easily some sections could be reshaped for mobile delivery. While the original source material was from the Mayo Clinic, which has been found broadly to have a rating of good for the ability of its patient-facing information to assist health care consumers on treatment information quality and resource validity [39], we have no validated measures that assess whether the original source material was effective. Finally, as this study focused solely on content characteristics and not on patient outcomes, no conclusions can be drawn about clinical effectiveness.

### Conclusions

This early report highlights the value of a human-centered, interdisciplinary process for adapting therapeutic materials for digital delivery within an mHealth app–based SUD treatment intervention. By combining clinical SUD expertise, lived experience in recovery, and health care communication knowledge through iterative drafting and review, the team produced content that, in aggregate, exceeded recommended PEMAT-P thresholds. Understandability was superior to actionability; at the module level, 35.7% (5/14) of modules fell below the acceptable threshold. This fits within the larger landscape of patient-facing materials, where actionability is often lacking. As digital SUD interventions continue to expand, leaving patients with clear actions that can be taken based on the content presented remains an important challenge. These findings also suggest that a robust adaptation process is not enough to guarantee strong content. Including active patients in this content design and iteration process may be beneficial to this end. Evaluating material at the unit level (eg, modules and chapters) instead of broadly as one corpus, may help ensure isolated weaknesses are not missed.

## Abbreviations

LADC: licensed alcohol and drug counselor
mHealth: mobile health
PEMAT-P: Patient Education Materials Assessment Tool for print materials
SUD: substance use disorder
